# Survival after Laparoscopic versus Abdominal Radical Hysterectomy in Early Cervical Cancer: A Randomized Controlled Trial

**DOI:** 10.31557/APJCP.2021.22.1.93

**Published:** 2021-01

**Authors:** Luciana Silveira Campos, Leo Francisco Limberger, Airton Teltebon Stein, José Manuel Caldas

**Affiliations:** 1 *Instituto de Saúde Pública da Universidade do Porto, Universidade do Porto, Portugal. *; 2 *Serviço de Ginecologia, Hospital Nossa Senhora da Conceição, Porto Alegre, Brazil. *; 3 *Departamento de Saúde Pública da Universidade Federal de Ciências da Saúde de Porto Alegre, Porto Alegre, Brazil. *; 4 *Serviço de Medicina de Família, Hospital Nossa Senhora da Conceição, Porto Alegre, Brazil. *

**Keywords:** Cervical cancer, laparoscopy, survival, randomized controlled trial

## Abstract

**Background:**

Previous studies have reported the safety of laparoscopic radical hysterectomy for treatment of early cervical cancer, as option to laparotomy. This study aims to compare overall survival between laparoscopic versus abdominal radical hysterectomy for early cervical cancer.

**Methods:**

A single-center randomized controlled trial enrolled 30 patients with clinically staged IA2 cervical cancer and lymphovascular invasion, IB and IIA, who underwent laparoscopic radical hysterectomy (16) or abdominal radical hysterectomy (14).

**Result:**

The mean overall survival time was 74.74 months (CI 95%: 54.15-95.33) for LRH 91.67 months (CI 95%: 74.97-108.37) for ARH (log-rank test = 0.30). The mean disease-free survival time was 81.07 months (CI 95%: 60.95-101.19) for LRH and 95.82 months (CI 95%: 80.18-111.47) for ARH (log-rank test = 0.371). The overall survival hazard ratio was 2.05 (CI 95%: 0.51-8.24), and the disease-free hazard ratio was 2.13 (CI 95%: 0.39-11.7).

**Conclusion:**

Our study suggests a non-significant trend of worse outcomes for LRH. In light of recent controversy and need for prospective studies, further studies in different populations are required for definite conclusions and until then, patients should be aware of risks and benefits, survival data and quality of life outcomes related to both surgical techniques.

## Introduction

Radical hysterectomy with pelvic lymphadenectomy is one of the recommended treatments by the International Federation of Gynecology and Obstetrics for early cervical cancer, which can be performed by abdominal, laparoscopic or robotic approaches (Ramirez et al., 2018).

Previous studies have reported the safety of laparoscopic radical hysterectomy (LRH) (Spirtos et al., 2002; Ramirez et al., 2006; Díaz-Feijoo et al., 2008). Systematic reviews comparing minimally invasive techniques (either laparoscopic or robotic) and abdominal radical hysterectomy (ARH) have been published (Cao et al., 2015; Wang et al., 2015; Jin et al., 2018). In a meta-analysis designed to evaluate surgical and prognostic factors, there was a longer operative time and lower estimated blood loss for LRH than for ARH. There was no difference in the number of pelvic nodes retrieved, and the results regarding perioperative and postoperative complications were controversial. Disease-free survival (DFS) and overall survival (OS) showed no differences (Cao et al., 2015).

In 2018, a large phase 3 randomized controlled trial comparing laparoscopic/robotic versus abdominal radical hysterectomy for early cervical cancer challenged the perceived oncological safety of LRH (Ramirez et al., 2018; Cohen et al., 2019). A total of 319 patients were submitted to LRH compared to 312 who underwent ARH, with a higher recurrence rate for those who received LRH (Ramirez et al., 2018). Some studies further described different results. A population-based study compared 475 LRH patients to 483 ARH patients operated on from 2006 to 2017 with a median follow-up of 6 years, adjusted for patient factors and surgeon volume, concluded that LRH was oncologically safe for IA but not IB tumors (Cusimano et al., 2019). A systematic review of 4731 patients in 17 studies that included only low- and middle-income countries described similar overall and DFS rates for LRH and ARH (Allanson et al., 2019).

A single-center randomized controlled trial of postoperative pain and other early outcomes of our group has already been published (Campos et al., 2013). The objective of the current study is to describe the results of long-term survival.

## Material and Methods

Detailed eligibility criteria, recruitment, endpoints, surgical techniques, anesthesia, analgesia, sample size calculation and randomization have been previously published (Campos et al., 2013). This study was a single-center randomized controlled trial comparing LRH with ARH in a tertiary public hospital in the southern region of Brazil. Eligible patients were randomized to undergo either LRH or ARH. We enrolled patients who underwent the operations from 1999 to 2004.

Women who were enrolled in the study were at least 18 years of age, had histologically confirmed primary squamous, adenocarcinoma or adenosquamous cervical cancer and staged according to the FIGO classification as IA2 with lymphovascular invasion, IB or IIA (Cohen et al., 2019). Patients with clinically advanced disease (IIB-IV), previous pelvic or abdominal radiotherapy, pregnancy or clinical diseases that would preclude one or both surgical approaches were excluded.

All surgeries were performed by the same team. LRH and ARH were performed according to the Piver III classification for radical hysterectomy (Piver et al., 1974; Spirtos et al., 1996). ARH access to the abdominal cavity was obtained through a vertical midline incision. Detailed surgical technique is described in the supplementary appendix. Adjuvant postoperative treatment was indicated based on histopatological findings according to the responsible physician decision. All patients were evaluated by the study team in the early postoperative period. The long-term follow-up by the study team or the personal physician was five years.

*Randomization and statistical analysis*

Patients were assigned to groups by a random number table of 180 five-digit numbers, which were generated by an author (ATS) who did not participate in the patient selection, surgery or follow-up. After informed consent was obtained and before the surgery was performed, a random allocation number was determined by a telephone call.

An intention-to-treat analysis was performed. Continuous variables with normal distribution were analyzed using Student´s t-test for independent samples and expressed as means and standard deviation, whereas nonnormally distributed variables were analyzed using the Mann-Whitney test and are expressed as medians and percentiles. Discrete variables were compared using Pearson’s chi-square test and Fisher´s exact test. DFS times were calculated in months from the date of surgery to the date of recurrence, censoring or the last follow-up exam. OS was calculated in months from the date of surgery to the date of death, censoring or the last follow-up exam. Survival curves and rates were calculated using the Kaplan-Meier method, and differences in survival times between groups were compared using the log-rank test. In Kaplan-Meier curves, deaths related to treatment were analyzed together with the deaths that were caused by the disease. Differences were considered statistically significant when the p values were < 0.05 in the two-sided test. The hazard ratio was the applied effect size. SPSS version 18.0 was used for the statistical analysis.

*Ethical considerations*

The Ethics Committee of Grupo Hospitalar Conceição approved the study protocol in 1999. This protocol was registered at ClinicalTrials.gov, Trial Registration: NCT01258413.

## Results

**Figure 1:**
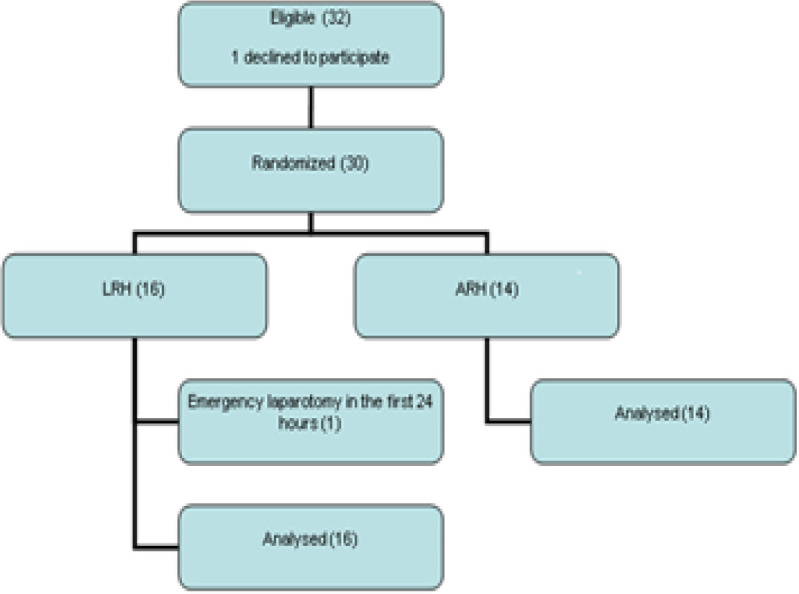
Study Flowchart

From 1999-2004, a total of 30 patients were included in this trial and underwent randomization. Sixteen patients were submitted to LRH, and 14 patients were submitted to ARH. No conversion to laparotomy occurred in the LRH group. (Figure 1) Three patients in both groups presented positive pelvic lymph nodes and all patients received adjuvant radiotherapy. At the time of the analysis, 6 LRH patients (37.5%) and 3 ARH (21.3%) patients had died (Fisher’s exact test = 0.44). In both groups, 2 patients died of causes not related to cervical cancer recurrence or metastasis. In the LRH group, one patient died due to the consequences of primary bladder cancer and another one died of breast cancer. In the ARH group, one patient died due to the consequences of bilateral ureteral stenosis after pelvic adjuvant radiotherapy. In the Kaplan-Meier analysis, this patient was included in the group of deaths related to cervical cancer. The second patient died of sepsis. In 4 patients who died of cervical cancer recurrence or metastasis, palliative chemotherapy was proposed. Three LRH patients (18.75%) and 1 ARH patient (7.14%) experienced pelvic recurrence. These patients palliative treatment and were alive at the time of the analysis (p = 0.602). All of the other patients were alive and free of disease.

**Figure 2:**
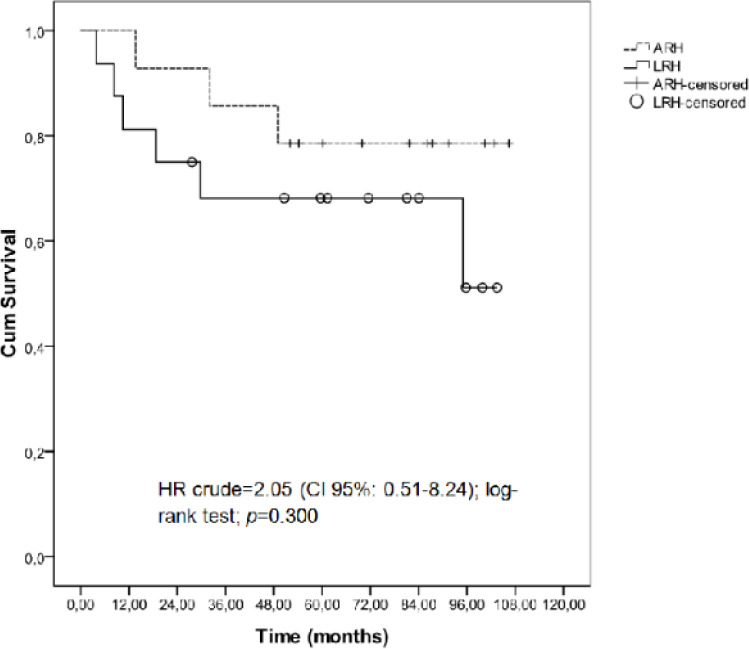
Overall Survival Rates in Early Cervical Cancer Patients who Underwent Laparoscopic Radical Hysterectomy or Abdominal Radical Hysterectomy

**Figure 3:**
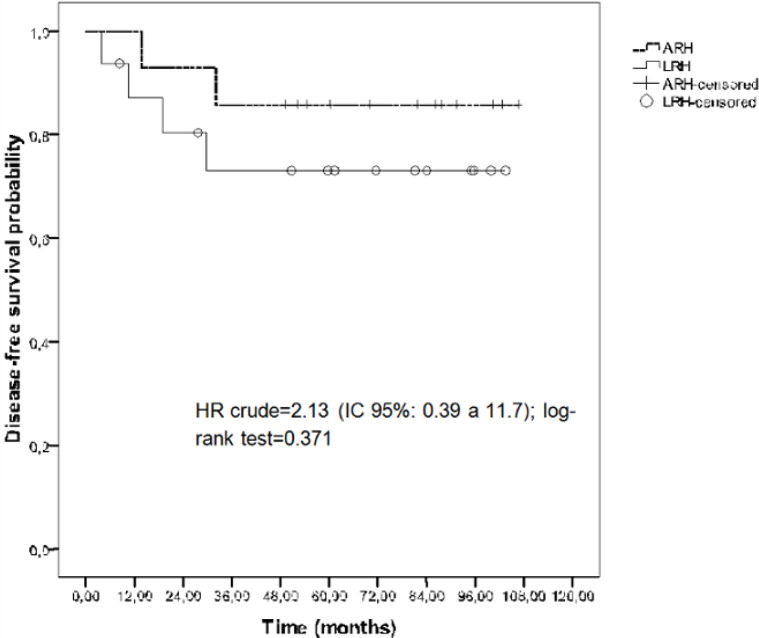
Disease-Free Survival Rates in Early Cervical Cancer Patients who Underwent Laparoscopic Radical Hysterectomy or Abdominal Radical Hysterectomy

The survival outcomes did not differ statistically between groups (Figures 2 and 3). The mean OS time was 74.74 months (CI 95%: 54.15-95.33) for the LRH group and 91.67 months (CI 95%: 74.97–108.37) for the ARH group (log-rank test = 0.30). The mean DFS time was 81.07 months (CI 95%: 60.95-101.19) for the LRH group and 95.82 months (CI 95%: 80.18-111.47) for the ARH group (log-rank test: 0.37). There were no significant differences in the OS or DFS between the groups. The 5-year OS rate was 68.2% for the LRH group and 78.6% for the ARH group (log-rank test = 0.30). The 5-year DFS rate was 73.1% for the LRH group and 85.7% for the ARH group (log-rank test: 0.37). The OS hazard ratio was 2.05 (CI 95%: 0.51-8.24), and the disease-free hazard ratio was 2.13 (CI 95%: 0.39-11.7).

## Discussion

In our study, LRH and ARH had equivalent survival rates, supporting some previous comparative studies (Díaz-Feijoo et al., 2008; Nam et al., 2012; Cao et al., 2015; Wang et al., 2016). A study compared IA2-IIB cervical cancer patients submitted to LRH (263) and ARH (263) who were matched for high-risk recurrence factors. There were no differences in the recurrence rate (HR = 1.28, CI 95%: 0.62-2.64) or death (HR = 1.46, CI 95%: 0.62-3.43) and the 5-year recurrence-free survival (RFS) rates for LRH and ARH patients were 92.8% and 94.4%, respectively (p = 0.499) (Nam et al., 2012). A matched cohort for surgicopathological risk factors for recurrence included 203 pairs of patients who received LRH or ARH. Both groups presented similar 5-year RFS rates (91.3% vs 90.4%, p = 0.83) and OS rates (93.2% vs 92.1%, p = 0.94) (Wang et al., 2016). A meta-analysis evaluated surgical morbidity and survival after LRH and included 4731 patients from 17 studies. The 5-year progression-free survival rate for LRH was 84% (n= 664; CI 95%: 0.81-0.87) and for ARH was 83% (n=3917; CI 95%: 0.75-0.89) (Allanson et al., 2019). However our mean differences IN OS of 16.3 months, 10.4%, and 2.05 fold, favoring ARH, and our mean differences in DFS of 14,75 months, 12.6%, and 2.13 fold, favoring ARH, could not be negligible.

This has been a matter of debate in the literature since 2018 (Melamed et al., 2018; Ramirez et al., 2018; Cusimano et al., 2019) The LACC trial, the first multicenter randomized controlled trial, which included patients with IA1 (lymphovascular invasion), IA2 and IB1 cervical cancer, compared 319 LRH and 312 ARH patients. LRH was associated with a lower DFS than that associated with ARH. Three-year rates were 91.2% and 97.1% for disease recurrence or death from cervical cancer (HR: 3.74, CI 95%: 1.63 to 8.5). Most patients in this study had 1B1 disease (Ramirez et al., 2018). A retrospective cohort of patients with IA2-IB1 cervical cancer from 2000 to 2013 from the Surveillance, Epidemiology End Results database (SEER) and the National Cancer Database (NCDB) included 1225 patients who received LRH and 1236 who received ARH. The 4-year mortality was 9.1% for LHR and 5.3% for ARH (HR: 1.65, CI 95%: 1.22-2.22) (Melamed et al., 2018). A population-based study compared 475 LRH patients to 483 ARH patients operated on from 2006 to 2017 with a median follow-up of 6 years, adjusted for patient factors and surgeon volume, concluded that LRH was associated with an increased rate of death (HR: 2.20, CI 95%: 1.15-4.19) and recurrence (HR: 1.97, CI 95%: 1.10-3.50) for IB tumors but not for IA (HR: 0.73, CI 95%: 0,13-4.01; HR: 0.34, CI 95%: 0.10-1.10) (Esteves et al., 2004).

The inclusion of tumors > 4 cm in our study reflected the regional practice pattern of that time, when the availability of brachytherapy was not widespread (Esteves et al., 2004) and that may contribute to the lower survival rate observed here. A noncontrolled study described a 20-year follow-up of 240 patients after LRH and reported that the 5-year survival rates for IA2, IB1, IB2, and IIA cervical cancer were 100%, 82%, 66%, and 60%, respectively (Yan et al., 2011). In most high-income country studies in the literature, patients underwent MRI and/or a CT (Ramirez et al., 2006; Díaz-Feijoo et al., 2008), however, they were not available in our hospital at that time. These exams could help to exclude patients with early parametrial invasion that can be underestimated by pelvic examination (Alvarez et al., 1991; Hoffman et al., 2004).

Our study suggests a non-significant trend of worse outcomes for LRH. However, our main limitation is the small sample size. After the LACC trial (Ramirez et al., 2018),the National Comprehensive Cancer Network (NCCN) recommended careful counseling the patients about short-term versus long-term outcomes and oncologic risks of the different surgical approaches (NCCN, 2019). The European Society of Gynaecologic Oncology also recommended that minimally invasive radical hysterectomy should be performed only by trained surgeons, the efforts be made to avoid spillage of tumor cells in the peritoneal cavity, that patients be informed about research data of the two surgical approaches 3 (ESGO, 2019) In light of recent controversy and need for prospective studies, we believe that our trial can contribute to the literature until a consensus is reached. Further studies in different populations are required for definite conclusions and until then, patients should be aware of risks and benefits, survival data and quality of life outcomes related to both surgical techniques.
